# Low-Complexity Joint Range and Doppler FMCW Radar Algorithm Based on Number of Targets

**DOI:** 10.3390/s20010051

**Published:** 2019-12-20

**Authors:** Bong-seok Kim, Sangdong Kim, Youngseok Jin, Jonghun Lee

**Affiliations:** Advanced Radar Technology Laboratory (ART Lab.), Daegu Gyeongbuk Institute of Science and Technology (DGIST), Daegu 42988, Korea; remnant@dgist.ac.kr (B.-s.K.); kimsd728@dgist.ac.kr (S.K.); ysjin@dgist.ac.kr (Y.J.)

**Keywords:** FMCW, low complexity, partial DFT, 2D FFT

## Abstract

A low-complexity joint range and Doppler frequency-modulated continuous wave (FMCW) radar algorithm based on the number of targets is proposed in this paper. This paper introduces two low-complexity FMCW radar algorithms, that is, region of interest (ROI)-based and partial discrete Fourier transform (DFT)-based algorithms. We find the low-complexity condition of each algorithm by analyzing the complexity of these algorithms. From this analysis, it is found that the number of targets is an important factor in determining complexity. Based on this result, the proposed algorithm selects a low-complexity algorithm between two algorithms depending the estimated number of targets and thus achieves lower complexity compared two low-complexity algorithms introduced. The experimental results using real FMCW radar systems show that the proposed algorithm works well in a real environment. Moreover, central process unit time and count of float pointing are shown as a measure of complexity.

## 1. Introduction

Recently, there have been several studies on radar sensors because they have many merits and can withstand external effects compared other sensors, such as weather and light. Therefore, radar sensors have been used as the primary sensors in several applications [1,2,3,4,5,6]. In radar sensors, continuous wave (CW) radars continuously transmit and receive electromagnetic waves, and the velocity and angular position are measured. However, the range of a target cannot be detected without employing additional modulation. Meanwhile, pulsed radars use a train of pulsed waveforms. These radars can detect the range of target and its velocity compared to CW radars. However, pulsed radars require very large bandwidth at the baseband [1,2,3].

Meanwhile, there have been several studies on frequency-modulated continuous wave (FMCW) radar systems as they have many advantages, such as low cost and low complexity [7,8,9,10,11,12,13]. Compared to pulsed radar, which requires large bandwidth and high cost, FMCW radar systems have relatively narrow bandwidth and low transmitted peak power, thus FMCW radar can meet certain range and velocity requirements with relatively low cost hardware and architectures [7,8,9,10,11,12,13]. In FMCW radar systems, a fast Fourier transform (FFT) is typically used as an estimator for parameters such as range, velocity, and angle. This is because the signals used in FMCW radar are sinusoidal. To detect multiple parameters, an FFT with a certain dimension is required depending on the parameters being determined. For example, a two-dimensional (2D) FFT is required to estimate the range and velocity, while a four-dimensional FFT is required to estimate range, velocity, azimuth, and elevation. Therefore, the complexity significantly increases as the number of dimensions in the FFT increases.

In [11], meanwhile, a low-complexity algorithm has been proposed by employing difference only two beat signals in order to effectively detect moving target. In the cases of stationary target and clutter, there is no Doppler effect. Hence, two beat signals are the same except for noise term and thus the difference of two beat signals contains only noise term. On the other hand, in the case of moving target, there is Doppler effect due to the moving target and thus the difference of them contains the range information of moving target. However, this algorithm might miss the moving target with certain velocity because this algorithm fixedly employs two beat signals. In order to overcome this disadvantage, an FMCW radar algorithm has been proposed by randomly employing two beat signals in [14]. This algorithm effectively avoids missing a target with a certain velocity by randomly selecting two beat signals every frame. In addition, this algorithm performs an angle detection algorithm only if there is a moving target. Therefore, this algorithm reduces the overall complexity. However, this algorithm has still a disadvantage in that it does not detect the velocity of the target. This is because the difference between the two beat signals is used to reduce the complexity of the moving target detection process and in this process, information necessary for the velocity detection of the target is lost.

Meanwhile, in [15,16,17,18], low-complexity detection algorithms for FMCW radar have been proposed which intend to reduce the number of FFTs compared to a full-dimension FFT-based FMCW radar algorithm. These algorithms determine a region of interest (ROI), thus reducing the number of inputs in the FFTs for Doppler estimation. However, there is still unnecessary computational complexity in these algorithms, although the complexity is reduced. The number of range bins used as the input in FFTs for Doppler estimation depends on the number of targets. In this algorithm, all chirp signals are used in an FFT to determine the range bins in which peaks exist. However, there is a disadvantage in that the number of range bins calculated in the first FFT for range estimation is too large compared to the number of range bins used as inputs in the FFT for estimation of the second parameter. In [19], in order to reduce the complexity, a low-complexity algorithm has been proposed by employing partial discrete Fourier transform (DFT). This algorithm performs Doppler FFT only on meaningful range bins, not on all the range bins. However, when the number of targets is small, the complexity may be lower than when the FFT is fully performed by partially performing the DFT only in the region where the target exists [20].

In this paper, we derive the required multiplications of low-complexity algorithms using ROI and partial DFT in order to compare complexity of two algorithms. From this analysis, it is found that the number of targets is an important factor in determining complexity. Therefore, depending on the estimated number of targets estimated by the range bins determined to be present among the range bins obtained through the range FFT, i.e., ROI detection, the overall complexity is reduced by selecting the lower complexity algorithm between full FFT and partial DFT algorithms. By doing so, the proposed algorithm further reduces the overall complexity compared to low-complexity algorithms that use an ROI. The proposed algorithm overcomes the shortcomings of [14], which cannot detect the velocity of the target because it uses all beat components corresponding to ROI. Simulation results show that the complexity according to the number of targets in order to check our derived criterion. Furthermore, experimental results with real FMCW radar systems show that the proposed algorithm works well in a real environment.

The structure of the paper is as follows. In Section 2, we introduce and define FMCW radar systems and the FMCW radar algorithm using a 2D FFT. Section 3 addresses the low-complexity FMCW radar algorithms, that is, ROI-based algorithm [15,16,17] and partial DFT-based algorithm [19]. We compare the complexity of two algorithms and find the condition of low complexity of each algorithm. Then, the proposed algorithm is addressed based on this condition. Experimental results involving 24 GHz FMCW radar systems in various cases are provided in Section 4 to verify the effectiveness of the proposed algorithm. Finally, the paper is concluded in Section 5.

## 2. System Model and Conventional Detection Algorithm Using 2D FFT

### System Model

This section addresses the system model of the conventional detection algorithm using a 2D FFT in FMCW radar systems. The key point of this paper is to reduce the unnecessary computational complexity of the FFT for parameter estimation. Therefore, we consider performing a fully 2D FFT for range and velocity detection for convenience. In this paper, angular detection is not considered because the FFT dimension would need to increase.

**Figure 1:**
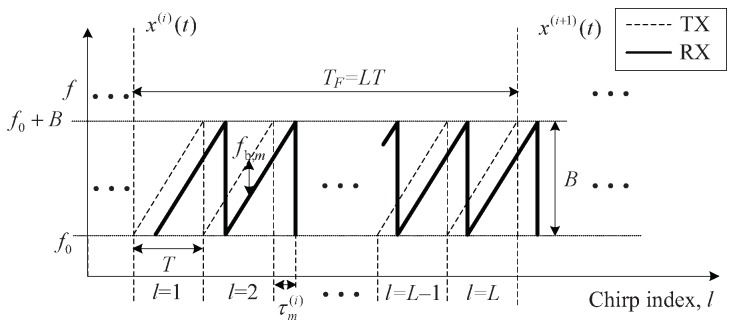
Structure of transmit and receive signals used with a frequency-modulated continuous wave (FMCW) radar.

Figure 1 shows that the transmitted (TX) FMCW signal in the *i*th frame for $1\leq i\leq N_{F}$, in which a total of *L* chirps are transmitted, is denoted $x^{\left(i\right)}\left(t\right)$ and is expressed as follows:

(1) $$\begin{array}{c} x^{\left(i\right)}\left(t\right)=\sum_{l=0}^{L-1}x_{0}\left(t-lT-iT_{F}\right) \end{array}$$

where $N_{F}$ is the number of frames, *T* is the duration of an FMCW chirp signal $x_{0}\left(t\right)$, and $T_{F}$ is the duration of a frame, i.e., $T_{F}=N_{F}T$, as shown in Figure 1. An FMCW chirp signal $x_{0}\left(t\right)$ is expressed as follows [9]:

(2) $$\begin{array}{c} x_{0}\left(t\right)=\exp\left(j2\pi \left(f_{0}t+\frac{\mu }{2}t^{2}\right)\right)\;\;\mathrm{for}\;\;0\leq t\leq T \end{array}$$

where $f_{0}$ is the carrier frequency and μ is the chirp slope, i.e., μ=B/T, where *B* is the the bandwidth of FMCW chirp signal.

Considering *M* targets, the receive (RX) signal from the *l*th chirp in the *i*th frame is denoted $r_{l}^{\left(i\right)}\left(t\right)$ and is expressed as follows [9]:

(3) $$\begin{array}{c} r_{l}^{\left(i\right)}\left(t\right)=\sum_{m=1}^{M}{\tilde{a}}_{m}^{\left(i\right)}x_{0}\left(t-\tau_{m}^{\left(i\right)}\right)v_{m}^{\left(i\right)}+{\tilde{w}}_{l,k}^{\left(i\right)}\left(t\right) \end{array}$$

where ${\tilde{a}}_{m}^{\left(i\right)}$ is the complex amplitude of the reflected signal from the *m*th target in the *i*th frame, $\tau_{m}^{\left(i\right)}$ is the time delay between the target and radar, and $v_{m}^{\left(i\right)}$ is a Doppler term due to the velocity of the *m*th moving target in the *i*th frame, i.e., $v_{m}^{\left(i\right)}=\exp\left(j2\pi f_{D,m}^{\left(i\right)}\left(Tl+\left(i-1\right)T_{F}\right)\right)$, ${\tilde{w}}_{l}^{\left(i\right)}\left(t\right)$ is an additive white Gaussian noise (AWGN) signal for the *l*th chirp and the *i*th frame. By multiplying the conjugated FMCW TX signal $x_{0}{\left(t\right)}^{*}$ by $r_{l}^{\left(i\right)}\left(t\right)$, the beat signal for the *l*th chirp in the *i*th frame $y_{l}^{\left(i\right)}\left(t\right)$ is obtained and expressed as the product of the range and velocity terms as follows:

(4) $$\begin{array}{ccc} y_{l}^{\left(i\right)}\left(t\right) & = & r_{l}^{\left(i\right)}\left(t\right)\times x_{0}{\left(t\right)}^{*}\\ & = & \sum_{m=1}^{M}a_{m}^{\left(i\right)}\underset{≜\eta_{m}^{\left(i\right)}\left(t\right)}{\underset{︸}{\exp\left(-j2\pi f_{b,m}^{\left(i\right)}t\right)}}v_{m}^{(i)l}+\underset{≜w_{l}^{\left(i\right)}\left(t\right)}{\underset{︸}{x_{0}{\left(t\right)}^{*}{\tilde{w}}_{l}^{\left(i\right)}\left(t\right)}}\\ & = & \sum_{m=1}^{M}a_{m}^{\left(i\right)}\eta_{m}^{\left(i\right)}\left(t\right)v_{m}^{(i)l}+w_{l}^{\left(i\right)}\left(t\right) \end{array}$$

where $a_{m}^{\left(i\right)}$ is the *m*th the complex amplitude term (except the range and velocity terms), which is defined as $a_{m}^{\left(i\right)}={\tilde{a}}_{m}^{\left(i\right)}\exp\left(-j(2\pi f_{0}\tau_{m}^{\left(i\right)}-\mu \tau_{m}^{(i)2}/2)\right)$ as in [21], and $f_{b,m}^{\left(i\right)}$ is the beat frequency, i.e., $f_{b,m}^{\left(i\right)}=\mu \tau_{m}^{\left(i\right)}$.

After performing analog to digital conversion (ADC) of $y_{l}^{\left(i\right)}\left(t\right)$, the discrete time model of (4) with sampling frequency $f_{s}$ is denoted by $y_{l}^{\left(i\right)}\left[n\right]$, i.e., $y_{l}^{\left(i\right)}\left[n\right]=y_{l}\left(n\left(T_{s}+\left(i-1\right)T_{F}\right)\right)$ for $n=0,1,\cdots ,N_{s}-1$, where $T_{s}=1/f_{s}$ is the sampling interval and $N_{s}=T/T_{s}$ is the number of samples. Therefore, the discrete time model in (4) can be rewritten as follows:

(5) $$\begin{array}{c} y_{l}^{\left(i\right)}\left[n\right]=\sum_{m=1}^{M}a_{m}^{\left(i\right)}\eta_{m}^{\left(i\right)}\left[n\right]v_{m}^{(i)l}+w_{l}^{\left(i\right)}\left[n\right]\;\;\mathrm{for}\;1\leq n\leq N_{S}. \end{array}$$

**Figure 2:**
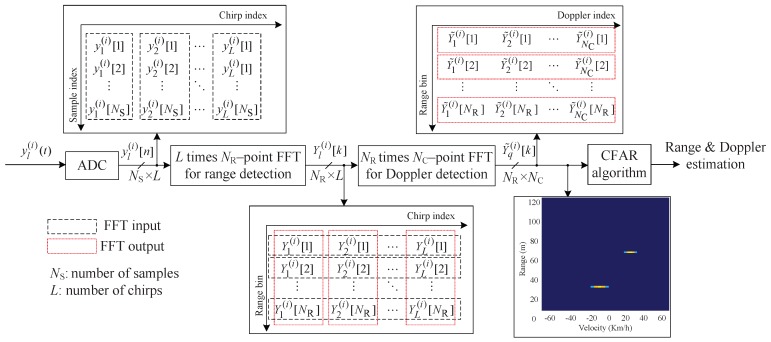
Structure of 2D fast Fourier transform (FFT) in FMCW radar.

From (5), the ADC beat signal can be expressed as a 2D sinusoidal signal, i.e., where *n* and *l* are the sample domain and the chirp domain, respectively. By performing 2D FFT on 2D sinusoidal signal, the sample and chirp domains are converted to the range and velocity (Doppler) domains, respectively. In other words, by estimating the frequencies of 2D sinusoidal signals in the sample and chirp domains, the desired parameters, i.e., the range and velocity of targets, can be detected. Figure 2 shows the structure of the parameter estimates using a 2D FFT during the *i*th frame. In Figure 2, $N_{R}$ and $N_{C}$ are the number of FFT points in the range and chirp domains, respectively. First, $N_{R}$ point FFTs is applied to the sample data obtained from the ADC $y_{l}^{\left(i\right)}\left[n\right]$ for 1≤l≤L to obtain range information. The *k*th FFT output of $y_{l}^{\left(i\right)}\left[n\right]$ is denoted $Y_{l}^{\left(i\right)}\left[k\right]$, i.e., the *k*th range bin is obtained for 1≤l≤L as follows:

(6) $$\begin{array}{c} Y_{l}^{\left(i\right)}\left[k\right]=\sum_{n=1}^{N_{s}}y_{l}^{\left(i\right)}\left[n\right]D_{N_{R}}^{\left(n-1)(k-1\right)}\;\;\mathrm{for}\;\;1\leq k\leq N_{R} \end{array}$$

where $D_{N}$ is the *N* point DFT operator, i.e., $D_{N}=\exp\left(-j2\pi /N\right)$. In other words, $N_{R}\times L$ range bins are obtained from (6). Second, in the same manner, $N_{C}$ point FFTs for Doppler estimation are applied to the $N_{R}\times L$ range bins, which is shown using the dashed line in Figure 2. The *q*th FFT output $Y_{l}^{\left(i\right)}\left[k\right]$ is denoted ${\tilde{Y}}_{q}^{\left(i\right)}\left[k\right]$ and is obtained for $1\leq k\leq N_{R}$ as follows:

(7) $$\begin{array}{c} {\tilde{Y}}_{q}^{\left(i\right)}\left[k\right]=\sum_{l=1}^{L}Y_{l}^{\left(i\right)}\left[k\right]D_{N_{C}}^{\left(l-1)(q-1\right)}\;\;\mathrm{for}\;\;1\leq q\leq N_{C}. \end{array}$$

As shown in Figure 2, the 2D FFT outputs reflect the range and Doppler (velocity) information of targets, which are obtained by stacking $N_{C}$ FFT outputs $N_{R}$ times for 1≤k≤K. As illustrated above, to estimate the range and velocity parameters, a full-dimensional FFT is performed on all range bins, regardless of the number of targets, which requires high computational complexity.

## 3. Low-Complexity FMCW Radar Algorithms

### 3.1. Low-Complexity FMCW Radar Algorithms Based on ROI

This section addresses the low-complexity range and Doppler estimation algorithm for FMCW radar systems compared to a 2D FFT by reducing the number of FFTs required for Doppler estimation [15,16,17]. The conventional 2D FFT algorithm mentioned in Section 2 has unnecessary complexity because full-dimensional FFTs are performed, regardless of the number of targets. In contrast, the low-complexity algorithm introduced in this section reduces the overall complexity by limiting the input of the FFT for Doppler estimates to range bins in which the targets are determined to exist, i.e., the region of interest (ROI), rather than all range bins. By doing so, this algorithm significantly reduces the computational complexity when estimating range and Doppler information.

**Figure 3:**
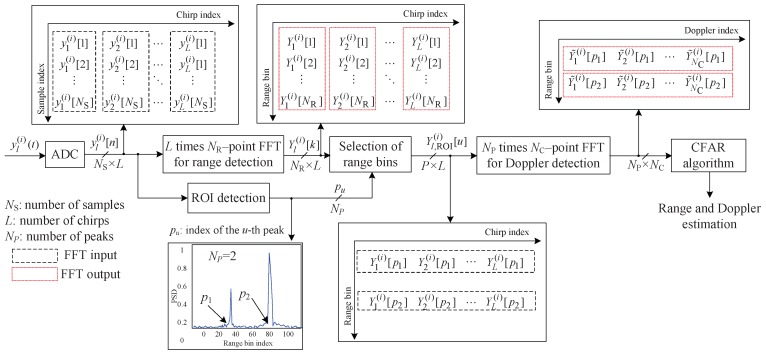
Structure of the low-complexity FMCW radar algorithm using region of interest (ROI).

**Figure 4:**
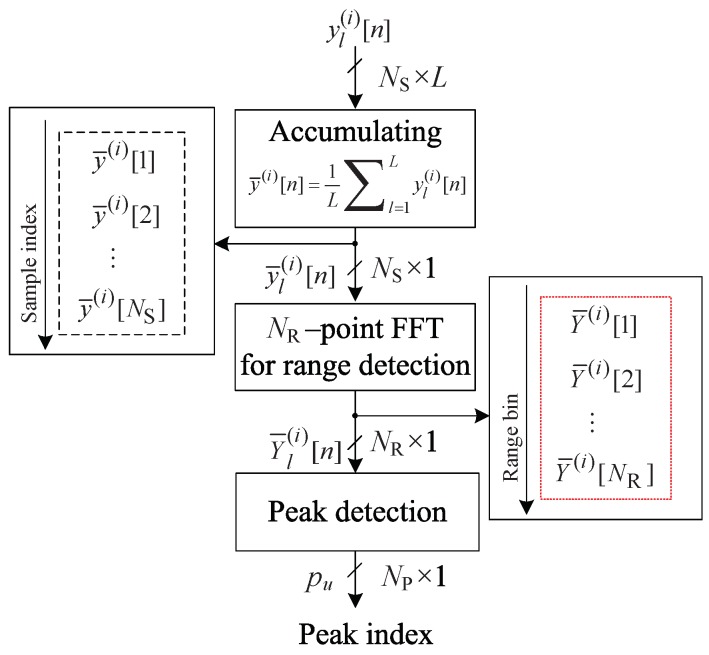
Structure of the ROI detection algorithm.

Figure 3 illustrates the structure of the low-complexity FMCW radar algorithm in the case of $N_{P}=2$, where $N_{P}$ is the number of output peaks from ROI detection. Compared to Figure 2, this algorithm and the 2D FFT algorithm are the same up to *L* time steps in $N_{R}$ point FFTs. In other words, from (5) to (6), a 2D FFT algorithm can also be used in this algorithm. After *L* time steps in $N_{R}$ point FFTs, in the low-complexity FMCW radar algorithm using an ROI, the ROI detection step is used to select only the range bins in which the targets are considered to be present. Figure 4 shows the structure of the ROI detection algorithm. To increase the reliability of ROI detection, the accumulated beat signal ${\bar{y}}^{\left(i\right)}\left[n\right]$ has been input to the FFT for range estimation, i.e., ${\bar{y}}^{\left(i\right)}\left[n\right]=1/N\sum_{l=1}^{L}y_{l}^{\left(i\right)}\left[n\right]$, as in [15,16]. Then, $N_{R}$ range bins are obtained by using an FFT on ${\bar{y}}^{\left(i\right)}\left[n\right]$ for range estimation. Peak detection is used to select $N_{P}$ range bins corresponding to the ROI in the $N_{R}$ range bins. The region of the range bins at a chirp that is $N_{R}$ in the 2D-FFT detection algorithm is reduced to $N_{P}$ in this algorithm. The *u*th reduced range bin in the ROI is denoted $Y_{l,\mathrm{ROI}}^{\left(i\right)}\left[u\right]$ and is expressed as follows:

(8) $$\begin{array}{c} Y_{l,\mathrm{ROI}}^{\left(i\right)}\left[u\right]=Y_{l}^{\left(i\right)}\left[u\right]\;\;\mathrm{for}\;\;1\leq u\leq N_{P}. \end{array}$$

This implies that the region *k* in (7) is modified as $k\in [p_{1},p_{2},\cdots ,p_{N_{P}}]$ instead of $1\leq k\leq N_{R}$, where $p_{u}$ is the index of the *u*th peak found in the ROI. Therefore, applying the *q*th Doppler FFT output to $Y_{l,\mathrm{ROI}}^{\left(i\right)}\left[u\right]$, denoted ${\tilde{Y}}_{q,\mathrm{ROI}}^{\left(i\right)}\left[u\right]$, is as follows:

(9) $$\begin{array}{c} {\tilde{Y}}_{q,\mathrm{ROI}}^{\left(i\right)}\left[u\right]=\sum_{l=1}^{L}Y_{l,\mathrm{ROI}}^{\left(i\right)}\left[u\right]D_{N_{C}}^{\left(l-1\right)\left(p_{u}-1\right)}\;\;\mathrm{for}\;\;1\leq q\leq N_{C}. \end{array}$$

In general, the number of the range bins in which the target exists $N_{P}$ is significantly smaller than the number of range bins $N_{R}$, thus the overall computational complexity is significantly reduced.

There is unnecessary computational complexity in this algorithm, although the complexity is significantly reduced compared to the conventional 2D FFT FMCW radar algorithm. The number of range bins used as input to the Doppler FFT is only *L* times $N_{P}$. However, to select *L* times $N_{P}$ range bins, it is necessary to obtain *L* times $N_{R}$ range bins. In general, because $N_{R}$ is significantly larger than $N_{P}$, there remains unnecessary complexity that can be further reduced.

### 3.2. Low-Complexity Algorithm Using Partial DFT

This section illustrates a low-complexity algorithm using partial DFT [19]. In ROI-based FMCW radar algorithm, *L* times $N_{R}$ point FFTs are performed in order to obtain *L* times $N_{p}$ range bins. On the other hand, in partial DFT-based FMCW radar algorithm, only $N_{p}$ partial DFTs are performed in order to obtain *L* times $N_{p}$ range bins instead of performing *L* times NR point FFTs.

ROI detection is conducted first in this algorithm, as was the case in the ROI-based algorithm. The range bin indices corresponding to the ROI are then determined. In order to perform a partial DFT with size $N_{R}$ corresponding to the ROI, a zero-padded beat signal $y_{l,\mathrm{ZP}}^{\left(i\right)}\left[n\right]$ is generated as follows:

(10) $$\begin{array}{c} y_{l,\mathrm{ZP}}^{\left(i\right)}\left[n\right]=\left\{\begin{array}{c} y_{l}^{\left(i\right)}\left[n\right],\;\;\mathrm{for}\;\;n=1,\;2,\cdots ,N_{S}\\ 0,\;\;\;\;\;\;\;\;\mathrm{for}\;\;n=N_{S}+1,\cdots ,N_{R}. \end{array}\right. \end{array}$$

Performing a partial DFT for 1≤l≤L yields the same $N_{P}\times L$ range bins as in (8) are obtained as follows:

(11) $$\begin{array}{c} Y_{l,\mathrm{ROI}}^{\left(i\right)}\left[u\right]=\sum_{n=1}^{N_{R}}y_{l,\mathrm{ZP}}^{\left(i\right)}\left[n\right]D_{N_{R}}^{\left(n-1\right)\left(p_{u}-1\right)}\;\;\mathrm{for}\;\;1\leq u\leq N_{P}. \end{array}$$

By employing partial DFT, overall complexity reduces compared to ROI-based algorithm. Of course, the complexity of a DFT is significantly higher than an FFT, thus the complexity of this algorithm using partial DFT might be higher than the low-complexity algorithm when the number of peaks $N_{P}$ and the number of FFT-points $N_{R}$ increases. Therefore, Section 3.3 compares the complexity of two low-complexity algorithms and identifies the optimal conditions for achieving low complexity.

### 3.3. Complexity Analysis

The complexity of each algorithm is compared in this section to analyze the conditions resulting in low complexity. Hereafter, we call the conventional 2D FFT-based algorithm the ‘2D FFT algorithm’, the low-complexity algorithm using an ROI the ‘ROI algorithm’, and the low-complexity algorithm using a partial DFT the ‘DFT algorithm’ for convenience. In addition, the required number of multiplications is used as a measure of complexity because the complexity involved in multiplication is high compared to other operations, such as addition and comparison.

The 2D FFT algorithm uses *L* times $N_{R}$ point FFTs for range estimation and $N_{R}$ times $N_{C}$ point FFTs for Doppler estimation, thus the required number of multiplications in the 2D FFT algorithm $C_{2D}$ is

(12) $$\begin{array}{c} C_{2D}=L\frac{N_{R}}{2}\log_{2}N_{R}+N_{R}\frac{N_{C}}{2}\log_{2}N_{C}. \end{array}$$

The ROI algorithm uses one $N_{R}$ point FFT to perform ROI detection, *L* times $N_{R}$ point FFTs for range estimation, and $N_{P}$ times $N_{C}$ point FFTs for Doppler estimation, thus the required number of multiplications in the ROI algorithm $C_{\mathrm{ROI}}$ is

(13) $$\begin{array}{c} C_{\mathrm{ROI}}=\left(L+1\right)\frac{N_{R}}{2}\log_{2}N_{R}+N_{P}\frac{N_{C}}{2}\log_{2}N_{C}. \end{array}$$

The DFT algorithm uses one $N_{R}$ point FFT to perform ROI detection and range estimation, $N_{P}$ times $N_{R}$ point DFTs to generate range bins and for range estimation, and $N_{P}$ times $N_{C}$ point FFTs for Doppler estimation, thus the required number of multiplications in the DFT algorithm $C_{\mathrm{DFT}}$ is

(14) $$\begin{array}{c} C_{\mathrm{DFT}}=\frac{N_{R}}{2}\log_{2}N_{R}+N_{P}LN_{R}+N_{P}\frac{N_{C}}{2}\log_{2}N_{C}. \end{array}$$

**Figure 5:**
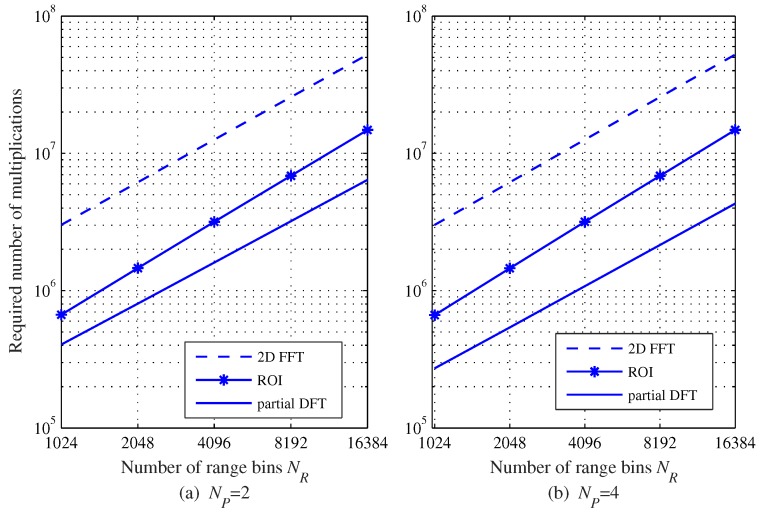
Required number of multiplications as a function of $N_{R}$ with $N_{C}=512$ and L=128; (**a**) $N_{P}=2$ and (**b**) $N_{P}=4$.

Figure 5 shows the required number of multiplications according to $N_{R}$ with $N_{C}=512$, L=128, and $N_{P}=2$ and $N_{P}=4$. Figure 5 shows that the required number of multiplications increases as $N_{R}$ increases in all algorithms. The 2D FFT algorithm has the highest complexity because this algorithm performs full 2D FFTs, regardless of the number of targets or the existence of a target. Meanwhile, the number of 2nd FFTs in the ROI algorithm, i.e., FFTs for Doppler estimation, decreases from $N_{R}$ to $N_{P}$, thus the overall complexity is significantly reduced compared to the 2D FFT algorithm. The DFT algorithm uses fewer FFTs for range and Doppler estimation, thus the overall complexity is further reduced compared to the ROI algorithm.

**Figure 6:**
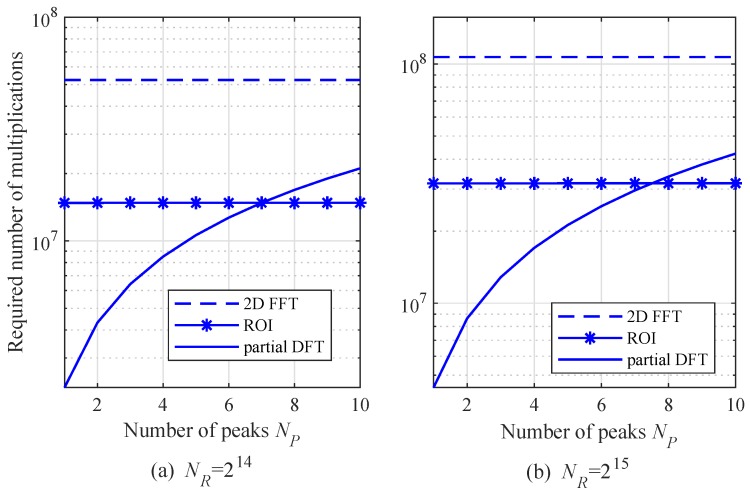
Required number of multiplications as a function of $N_{P}$ with $N_{R}=2^{14}$ and $2^{15}$, $N_{C}=512$, and L=128.

Figure 6 shows the required number of multiplications as a function of $N_{P}$ with $N_{R}$ = 16,384, $N_{C}=512$, and L=128. Note that the required multiplications in the proposed algorithm begins to exceed the number in the ROI algorithm when $N_{P}$ is greater than 7. As mentioned before, the complexity of the partial DFT algorithm might be larger than that in the ROI algorithm due to the high complexity of a DFT compared to an FFT. Hence, we focus on comparing the complexities of these two algorithms.

In order to identify the conditions resulting in low complexity, we determine a condition where the complexity of two algorithms becomes equal by calculating the difference between the complexities of the two algorithms, i.e., $C_{\mathrm{DFT}}=C_{\mathrm{ROI}}$. >From (13) and (14), the condition for $C_{\mathrm{DFT}}=C_{\mathrm{ROI}}$ is expressed using $N_{P}$ as follows:

(15) $$\begin{array}{c} N_{P}=\frac{1}{2}\log_{2}N_{R}. \end{array}$$

From (15), one concludes that the complexities of the two algorithms are equal when $N_{P}=\frac{1}{2}\log_{2}N_{R}$. This implies the following: if $N_{P}$ is less than $\frac{1}{2}\log_{2}N_{R}$ after ROI detection, it is more efficient to use the DFT algorithm. However, the ROI-based algorithm is better than the partial-DFT-based algorithm when $N_{P}$ is greater than $\frac{1}{2}\log_{2}N_{R}$.

### 3.4. Proposed FMCW Radar Algorithm with Further Reduced Complexity

**Figure 7:**
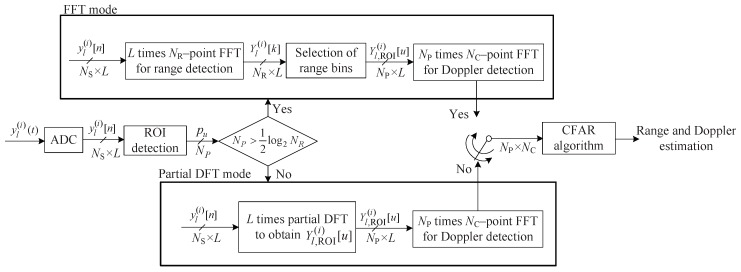
Structure of the proposed algorithm.

The proposed algorithm selects one between two algorithm, i.e., ROI-based and partial DFT-based algorithms in order to further reduced complexity. Figure 7 shows the structure of the proposed algorithm. As shown in Figure 7, the proposed algorithm first, checks the considering number of range bins, i.e., $N_{P}$ by ROI detection. Then, depending on $N_{P}$, one of two modes is employed. If $N_{P}>\frac{1}{2}\log_{2}N_{R}$, ROI-based algorithm is employed. On the other hand, $N_{P}\leq \frac{1}{2}\log_{2}N_{R}$, partial DFT-based algorithm is used.

## 4. Experiment Results

An experiment for verifying the effectiveness of the proposed algorithm in a practical environment is discussed in this section. First, the equipment and several experimental conditions are discussed. Second, the experimental results produced with the proposed algorithm are analyzed.

### 4.1. Experimental Setup

**Figure 8:**
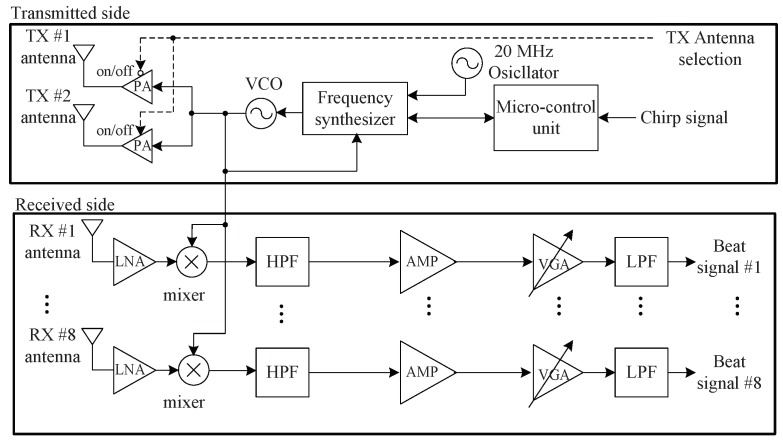
Block diagram of the 24 GHz radar module [14].

**Figure 9:**
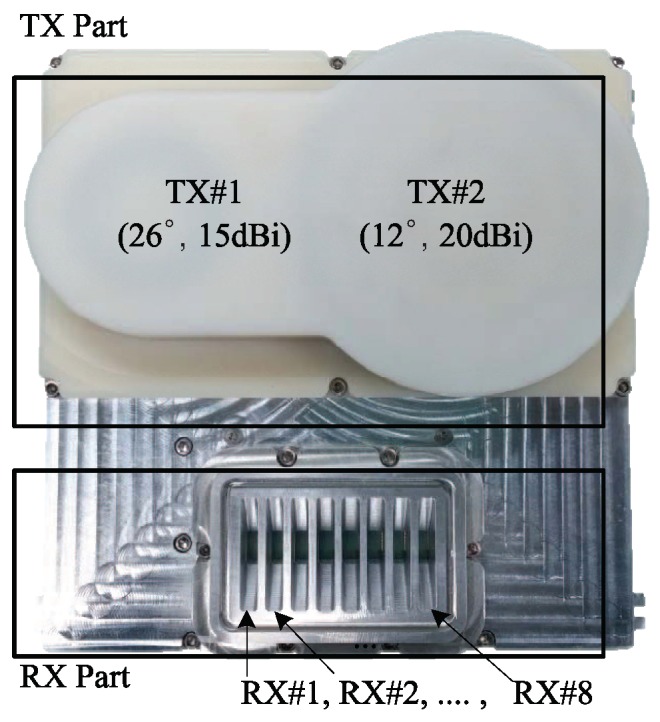
Image of 24 GHz front end module [21].

We used a 24 GHz FMCW radar system with two TX and eight RX antennas, as was used in [14,21]. Figure 8 shows a block diagram of the radio frequency (RF) front-end module i.e., the front end module (FEM) used in the experiment. Figure 9 shows an image of the FEM. The RF module was composed of TX and RX portions. A micro controller unit (MCU), frequency synthesizer with a phase-locked loop (PLL), and voltage-controlled oscillator (VCO) were included in the TX side. The MCU controlled the frequency synthesizer with the PLL to properly synchronize TX and RX channels. The VCO generated chirp signals with a frequency determined by the applied voltage, and its output was connected to the two TX antennas through a power amplifier. One TX antenna (dashed line in Figure 8) was used in this system. There were the eight RX antennas on the RX side with low noise amplifiers (LNAs), high pass filters (HPFs), amplifiers (AMP), a variable gain amplifier (VGA), and low pass filters (LPFs). The outputs from the LNAs were multiplied by the TX signals and are passed through the HPFs with 150 kHz bandpass frequency. An HPF was used to remove the DC-offset component from the direct conversion receiver in the FMCW radar system. The outputs from the HPFs were amplified using AMPs and VGAs, and the beat signal in each channel was obtained after the amplified signals are passed through the LPFs.

**Figure 10:**
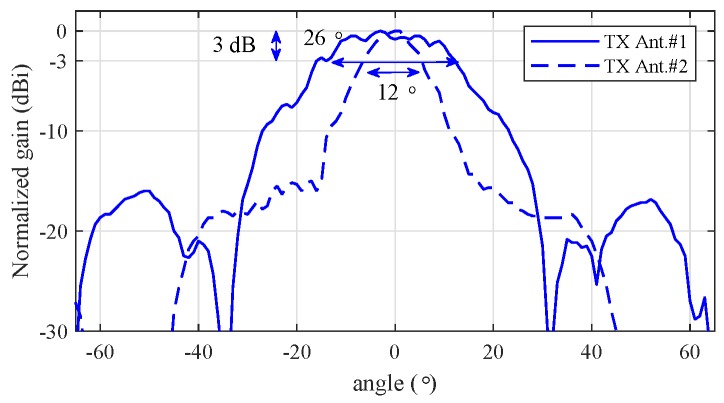
Radiation pattern of two transmitted antennas [21].

Figure 10 shows the normalized gain as a function of angle to determine the radiation pattern from the two TX antennas in the RF system. Figure 10 shows that the azimuth angles of the TX antennas corresponding to the beam-width with 3 dB gain are 26${}^{\circ }$ and 12${}^{\circ }$. This result implies that the first and second TX antennas can cover an azimuth angle of 26${}^{\circ }$ and 12 ${}^{\circ }$, respectively. The first TX antenna was used in this experiment.

**Figure 11:**
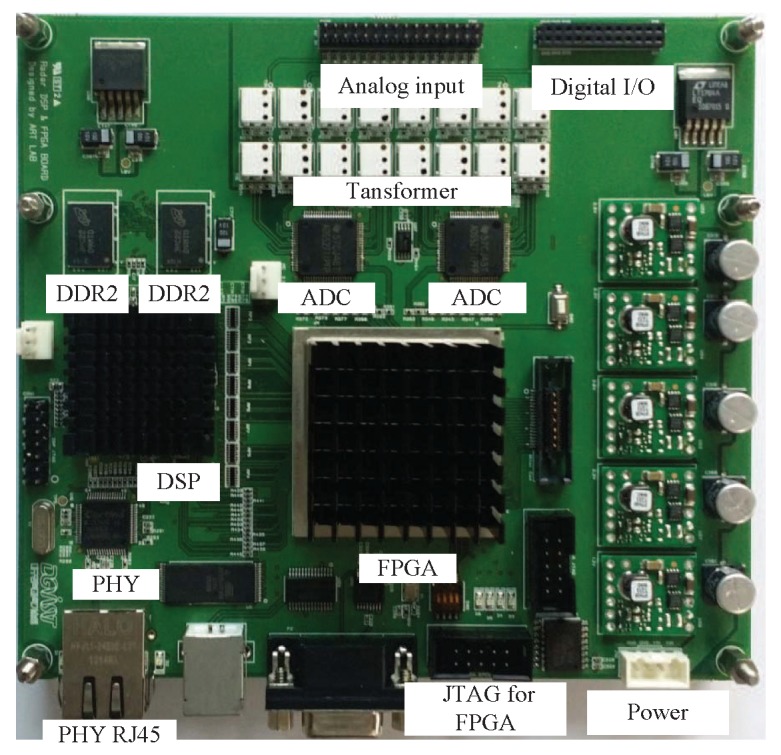
Data logging board used in the experiment.

**Figure 12:**
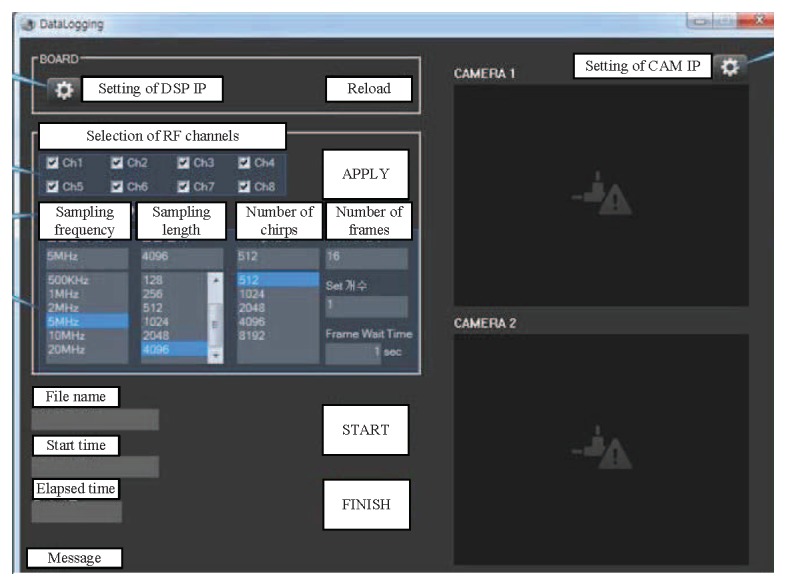
Graphic user interface (GUI) for the FMCW radar system.

Figure 11 and Figure 12 show the back end module (BEM) used in this system. The BEM contains a data logging board that includes digital signal processing capabilities, a field-programmable gate array, and graphic user interface (GUI) software for setting parameters for the logging board. The beat signals in eight channels from the analog input were converted to digital signals by the ADC with 20 MHz sampling rate. Two 2 GB DDR2 DRAM chips were used for external memory, providing a total of 512 Mbytes of data storage space. If the external memory was filled, the data was transferred to a computer via Ethernet (PHY RJ45). Figure 12 shows the GUI that provides the convenience of changing the logging board settings. Parameters such as the desired RF channel, sampling frequency, sampling length, number of chirps, and number of frames could be set in the GUI. Moreover, users can easily start and terminate the BEM system, as well as choose an IP address for communication with a PC. The channels to receive are selected in “Selection of RF channels”. The sampling frequency to be used in the ADC (500 kHz to 20 MHz) could be selected in “Sampling frequency”. The number of samples (up to 4096) could be chosen in “Sampling length”. The number of FMCW symbols to be transmitted (512 to 8192) could be selected in “Number of chirps”.

### 4.2. Experiment Analysis

**Figure 13:**
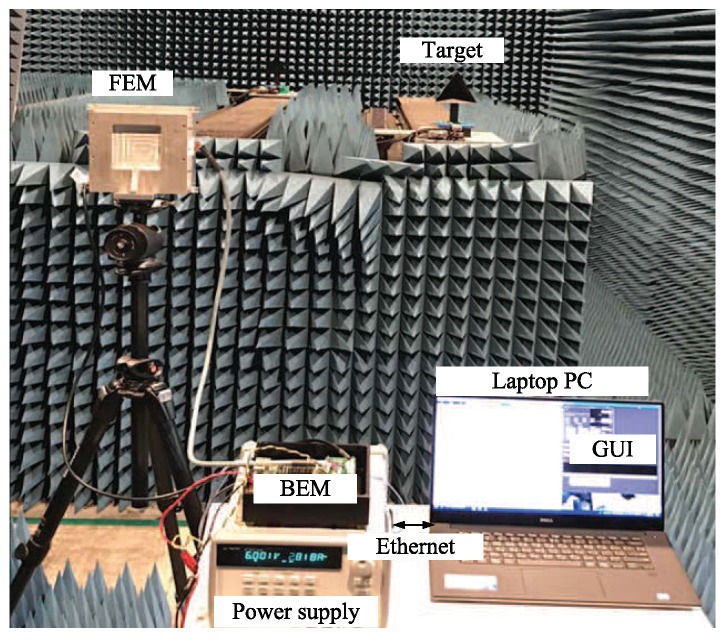
A photograph of the experimental set up in an anechoic chamber [21].

Figure 13 shows a real image of an experiment. To suppress the negative effects due to undesired echoes, the experiment was performed in an anechoic chamber located at the DGIST in Korea. This chamber is designed for use with 8 to 110 GHz frequencies, and its size is 5 m (W) × 10 m (L) × 4 m (H) [14]. Corner reflectors with 14 cm side length were used as targets to preserve the radar cross-section. 2048 point FFTs were performed for range estimation and 512 point FFTs were performed for Doppler estimation. The experimental parameters are shown in Table 1 [14]. The center frequency was set to 24 GHz, the bandwidth was set to 1 GHz, and the sampling frequency was set to 5 MHz. The duration of the chirp (ramp) *T* was set to 400 μs, the number of chirps per frame was set to 256, and the number of frames was set to 32.

**Figure 14:**
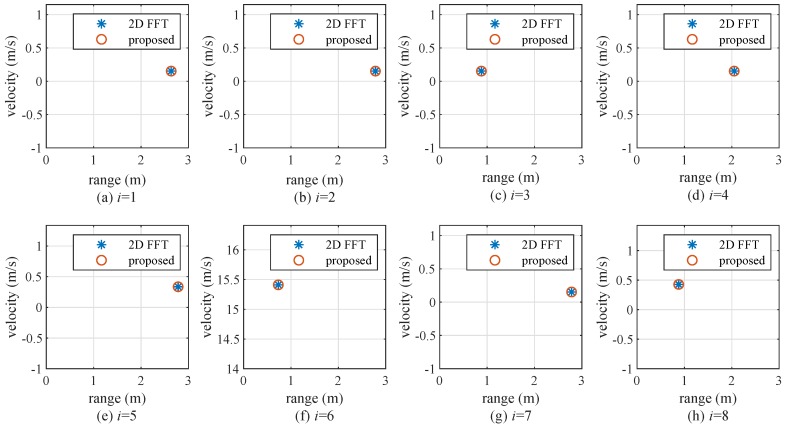
Experiment results of 2D FFT and the proposed algorithm at each frame (i= 1, 2,..., 8).

Figure 14 shows experimental results with a 2D FFT and the proposed algorithm from the 1st frame to the 8th frame. One can see that the proposed algorithm provides the same detection results as a 2D FFT at each frame, despite its very low complexity. These results confirm the effectiveness of the proposed algorithm.

**Figure 15:**
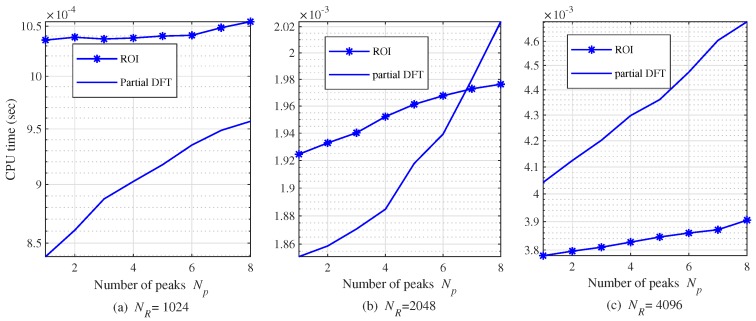
CPU time according to $N_{P}$ and $N_{R}$ with $N_{C}=512$ and L=128; (**a**) $N_{R}=1024$, (**b**) $N_{R}=2048$ and (**c**) $N_{R}=4096$.

Figure 15 shows central processing unit (CPU) time according to $N_{P}$ for several $N_{R}$s in order to quantitatively compare the computational complexity of two low-complexity algorithms. The number of FFT points for Doppler is set to 512 and the number of chirp signal, *L* is set to 128. In the case of $N_{R}=1024$, CPU time of partial-DFT based algorithm is lower compared to ROI-based algorithm for all $N_{P}$s. In the case of $N_{R}=2048$, it can be observed that CPU times of two algorithms cross each other when $N_{P}=7$. On the other hand, $N_{R}=4096$, ROI-based algorithm requires a lower CPU time compared to partial DFT-based algorithm for all $N_{P}$s.

**Figure 16:**
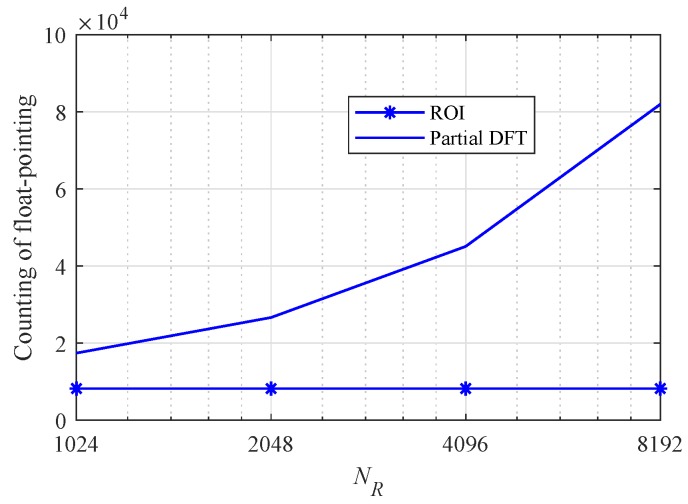
Counting of float pointing according to $N_{R}$ with $N_{P}=8$, $N_{C}=512$ and L=128.

Figure 16 shows counting of float pointing according to $N_{R}$ with $N_{p}=8$, as another measure of computational complexity. The number of FFT points for Doppler is set to 512 and the number of chirp signal, *L* is set to 128. Counting of float-pointing of ROI-based algorithm is the same regardless of $N_{R}$. On the other hand, counting of float-pointing of partial DFT-based algorithm gradually increases according to $N_{R}$.

## 5. Conclusions

We found the low-complexity condition of two low-complexity algorithms for FMCW radar by analyzing their complexity. In addition, it is found that the number of targets was an important factor in determining complexity. By experimental results, the proposed algorithm well detected range and Doppler despite low complexity. In order to compare the practical complexity of two algorithms, CPU time and the counting of float pointing were shown.

## Figures and Tables

**Table 1:** Experimental parameters [14].

Parameter	Value
Center frequency, $f_{0}$	24 GHz
Bandwidth, *B*	1 GHz
Chirp duration, *T*	400 μs
Number of chirps per one frame, *L*	256
Number of frames, $N_{F}$	32
Sampling frequency, $f_{s}$	5 MHz

## References

[1] Mahafza B.R. (2013). Radar Systems Analysis and Design Using MATLAB.

[2] Richards M.A. (2005). Fundementals of Radar Signal Processing.

[3] Levanon N. (2004). Radar Signals.

[4] Skolnik M.I. (2001). Introduction to Radar Systems.

[5] Jiang D., Liu M., Gao Y., Gao Y., Fu W., Han Y. (2018). Time-matching random finite set-based filter for radar multi-target tracking. Sensors.

[6] Zhang T., Sarrazin J., Valerio G., Istrate D. (2018). Estimation of human body vital signs based on 60 GHz Doppler radar using a bound-constrained optimization algorithm. Sensors.

[7] Stove A.G. (1992). Linear FMCW radar techniques. IEE Proc. Rad. Signal Process..

[8] Dudek M., Nasr I., Bozsik G., Hamouda M., Kissinger D., Fischer G. (2015). System analysis of a phased-array radar applying adaptive beam-control for future automotive safety applications. IEEE Trans. Veh. Tech..

[9] Saponara S., Neri B. (2017). Radar sensor signal acquisition and multidimensional FFT processing for surveillance applications in transport systems. IEEE Trans. Instrum. Meas..

[10] Matthew A., Matthew R., Kevin C. (2018). On the application of digital moving target indication techniques to short-range FMCW radar data. IEEE Sens. J..

[11] Jin Y., Kim B., Kim S., Lee J. (2018). Design and implementation of FMCW surveillance radar based on dual chirps. Elektron. Ir Elektrotechnika.

[12] Belyaev E., Molchanov P., Vinei A., Koucheryavy Y. (2013). The use of automotive radars in video-based overtaking assistance applications. IEEE Trans. Intell. Transp. Syst..

[13] Pan X., Xiang C., Liu S., Yan S. (2019). Low-complexity time-domain ranging algorithm with FMCW sensors. Sensors.

[14] Kim B., Jin Y., Kim S., Lee J. (2018). A low-complexity FMCW surveillance radar algorithm using two random beat signals. Sensors.

[15] Kim S., Oh D., Lee J. (2015). Joint DFT-ESPRIT estimation for TOA and DOA in vehicle FMCW radars. IEEE Antennas Wirel. Propag. Lett..

[16] Liu Y., Li Z.G., Soh Y.C. (2008). Region-of-interest based resource allocation for conversational video communication of H.264/AVC. IEEE Trans. Circuits Syst. Video Technol..

[17] Dehkordi S., Appenrodt N., Dickmann J., Waldschmidt C. Region of interest based adaptive high resolution parameter estimation with applications in automotive radar. Proceedings of the 2018 19th International Radar Symposium (IRS).

[18] Khan W., Qureshi I.M., Basit A., Khan W. (2016). Range-bins-based MIMO frequency diverse array radar with logarithmic frequency offset. IEEE Antennas Wirel. Propag. Lett..

[19] Chan H.C. (1994). Evaluation of the FMICW Waveform in HF Surface Wave Radar Applications.

[20] Haykin S. (1993). Radar Array Processing.

[21] Kim B., Kim S., Lee J. (2018). A novel DFT-based DOA estimation by a virtual array extension using simple multiplications for FMCW radar. Sensors.

